# Role of *CYP1B1*, *MYOC*, *OPTN* and *OPTC* genes in adult-onset primary open-angle glaucoma: predominance of *CYP1B1* mutations in Indian patients

**Published:** 2007-04-30

**Authors:** Arun Kumar, Manjunath G. Basavaraj, Santosh K. Gupta, Imteyaz Qamar, Abdullah Mahmood Ali, Vineeta Bajaj, T.K. Ramesh, D. Ravi Prakash, Jyoti S. Shetty, Syril K. Dorairaj

**Affiliations:** 1 Department of Molecular Reproduction, Development and Genetics, Indian Institute of Science, Bangalore, India; 2 Minto Ophthalmic Hospital, Bangalore, India; 3 Bangalore West Lions Superspecialty Eye Hospital, Bangalore, India

## Abstract

**Purpose:**

Mutations in the *CYP1B1*, *MYOC*, *OPTN*, and *WDR36* genes result in glaucoma. Given its expression in the optic nerve, it is likely a mutation in the *OPTC* gene is also involved in initiating glaucoma. This study was designed to evaluate the involvement of the *CYP1B1*, *MYOC*, *OPTN*, and *OPTC* genes in the etiology of adult-onset primary open-angle glaucoma (POAG) found in 251 Indian patients.

**Methods:**

Blood samples were obtained from individuals for DNA isolation. A combination of polymerase chain reaction-single strand conformation polymorphism, allele-specific PCR, and DNA sequencing techniques were used to detect mutations in four genes. Four microsatellite markers from the *CYP1B1* candidate region and three intragenic *CYP1B1* single nucleotide polymorphisms (SNPs) were used to determine the origin of the most common *CYP1B1* mutations.

**Results:**

Three previously known mutations (Pro193Leu, Glu229Lys, and Arg368His) and one novel (Met292Lys) mutation were found in the *CYP1B1* gene. Frequencies of the most common mutations, Glu229Lys and Arg368His, in patients were 5.12% and 3.98%, respectively. The Glu229Lys and Arg368His mutations were also found in normal controls at frequencies of 5% and 2%, respectively, suggesting that these mutations might be polymorphic variants in our population. The absence of allele sharing for D2S177, D2S1346, D2S2974, and D2S2331 markers and three intragenic *CYP1B1* SNPs in patients suggested multiple origins for the Glu229Lys and Arg368His variants. Two of 251 (0.8%) patients had the Gln48His mutation in *MYOC*. There was no difference in the frequency of a *MYOC* -83G>A promoter polymorphism between patients and controls. A novel *OPTN* mutation, Thr202Arg, was detected in one of 251 (0.4%) patients. The *OPTN* variant Met98Lys was detected in similar frequencies in patients and controls. No mutation was detected in *OPTC*. Taken together, 3.59% (9/251) of our POAG patients had mutations in the *CYP1B1*, *MYOC*, and *OPTN* genes.

**Conclusions:**

This is the first report to document the involvement of the *CYP1B1*, *MYOC*, and *OPTN* genes in the etiology of POAG in the same set of Indian patients. Our study shows that mutations in these genes are rare in Indian POAG patients.

## Introduction

Glaucoma is the leading cause of irreversible blindness and the second leading cause of blindness after cataract, affecting 66 million people worldwide [1]. Glaucoma is a heterogeneous group of progressive optic neuropathies characterized by an excavation of the optic disc and progressive alteration of the visual field [2]. High intraocular pressure (IOP), defined as being above 21 mmHg, in both eyes and a positive family history for glaucoma are commonly associated risk factors. Based on the age of onset and other clinical features, glaucoma has been classified into primary congenital glaucoma (PCG), juvenile-onset open-angle glaucoma (JOAG), and adult-onset primary open-angle glaucoma (POAG). PCG manifests at birth or in early infancy (up to 3 years of age). Its phenotype is characterized by elevated IOP, corneal edema, enlargement of the globe (buphthalmos), epiphora, photophobia, and blepharospasm. PCG occurs as a result of developmental anomalies of the anterior chamber angle that prevent drainage of the aqueous humor, thereby elevating IOP. PCG is most commonly inherited as an autosomal recessive trait. JOAG is characterized by early onset (at 10-35 years of age) and autosomal dominant inheritance with high penetrance. POAG occurs after the age of 35 years and is the most common form of glaucoma.

In a majority of cases, glaucoma does not follow a clear-cut inheritance pattern. However, clustering of multiple affected individuals within families suggests that it has a genetic basis. Linkage analyses have identified 23 loci (GLC1A-GLC1L, GLC3A-GLC3B, 2p14, 2q33-q34, 5q22.1-q32, 10p12-p13, 14q11, 14q21-q22, 17p13, 17q25, and 19q12-q14) for different forms of glaucoma [3-10]. However, only four genes (*MYOC*/*TIGR*, *CYP1B1*, *OPTN*, and *WDR36*) have been identified so far [4,11-13]. The myocilin/trabecular meshwork-induced glucocorticoid response protein (*MYOC*/*TIGR*) gene, located at the GLC1A locus on chromosome 1q24.3-q25.2, has been shown to cause glaucoma in 2-4% of POAG cases [14]. The cytochrome P450 (*CYP1B1*) gene, located at the GLC3A locus on chromosome 2p22-p21, has been shown to cause PCG, JOAG, and POAG [2,12,15]. In a large family in which *MYOC*-linked POAG segregated, a heterozygous mutation in *CYP1B1* was associated with early onset of the disease, indicating that a *CYP1B1* mutation might behave as a modifier of the *MYOC* gene [15]. The optineurin (*OPTN*) gene, located at the GLC1E locus on chromosome 10p14-p15, has been shown to cause normal tension glaucoma (NTG), a subtype of POAG [13]. The WD repeat-containing protein 36 (*WDR36*) gene, located at the GLC1G locus on chromosome 5q21.3-q22.1, has been shown to be mutated in POAG [4].

Glaucoma is a treatable disease if detected early. Therefore, development of an accurate test for the detection of presymptomatic carriers at risk is important for the management of glaucoma. To this end, a few studies have been carried out to assess the roles of the *MYOC* [16-19], *CYP1B1* [20], and *OPTN* [21,22] genes in the etiology of Indian POAG patients from different parts of the country. We have reported the genetic analysis of glaucoma in a large south Indian pedigree [18]. Moreover, there is no comprehensive study published to date that assesses the roles of three known glaucoma-causing genes (*CYP1B1*, *MYOC*, and *OPTN*) in the same set of Indian POAG patients. In this study, we report mutation analysis of the *CYP1B1*, *MYOC*, and *OPTN* genes in 251 Indian POAG patients from the south Indian state of Karnataka.

The *OPTC* (opticin) gene encodes a protein that is a member of the small leucine-rich repeat protein (SLRP) family, and is located on chromosome 1q31-q32 within an age-related macular degeneration (AMD) susceptibility locus [23]. Because of its protein profile in different parts of the eye, such as the iris, trabecular meshwork/ciliary body, retina, vitreous, and optic nerve, Friedman et al. [23] screened *OPTC* for mutations in individuals with POAG, NTG, and AMD. They failed to find any mutation in this gene. To rule out the *OPTC* gene as a glaucoma gene, we screened this gene in our POAG data set. The results of our analysis are presented herein.

## Methods

### Patients

A total of 251 patients with adult-onset POAG were evaluated at the Minto Ophthalmic Hospital, and Bangalore West Lions Superspecialty Eye Hospital. Both hospitals are located in the city of Bangalore, Karnataka. The patients were natives of Karnataka and spoke the south Indian Kannada language. They ranged in age from 45 to 65 years. Of the 251 patients, 116 patients received the diagnosis of POAG based on the presence of characteristic glaucomatous optic neuropathy and defects in visual fields. The remaining 135 patients were given the diagnosis of glaucoma based on glaucomatous optic neuropathy only, as visual field defects could not be assessed in these patients due to an advance stage of glaucoma. IOP was more than 21 mmHg in 198 patients. The remaining 53 patients had NTG with IOP below 21 mmHg and significant optic disc damage and visual field defects at the time of diagnosis. All patients had open-angles (Shafer grade greater than III) on gonioscopy and no other eye or systemic abnormalities. Patients with secondary causes of glaucoma (e.g., uveitis, steroid-induced, or trauma) were excluded from this study. We recruited 100 normal controls, also from Karnataka and with the same linguistic background, through several eye care camps. Their ages ranged from 45 to 75 years. All participants went through a detailed clinical examination and were found to have no signs or symptoms of glaucoma or any other eye disease. Controls did not have any family history of glaucoma. Informed consent was obtained from each patient. This study followed the guidelines of the Indian Council of Medical Research, New Delhi.

### Molecular study

Three to five ml of peripheral blood was collected from each individual in Vacutainer EDTA^TM^ tubes (Beckton-Dickinson, Franklin Lakes, NJ). Genomic DNA was isolated from blood samples using a Wizard® genomic DNA extraction kit (Promega, Madison, WI). Mutation analyses of the four genes were carried out using a combination of PCR-SSCP (polymerase chain reaction-single strand conformation polymorphism) and DNA sequencing techniques. For PCR-SSCP, primer sets were designed for the *CYP1B1*, *MYOC*, *OPTN*, and *OPTC* genes, which covered their entire coding regions and intron-exon junctions. The reference mRNA sequences for the *CYP1B1*, *MYOC*, *OPTN*, and *OPTC* genes used were U03688, NM_000261, AF420371, and NM_014359, respectively. The genomic sequences for these genes were retrieved from the UCSC Genome Bioinformatics site. Primer details are shown in Table 1 Since abnormalities in *WDR36* alone are not sufficient to cause POAG [24], we have not screened this gene for mutations in our POAG samples. In the future, we plan to screen this gene for mutations in our samples. PCR was carried out in a 25 μl reaction volume containing 50-100 ng of genomic DNA, 50 ng of each primer, 0.25 μl of alpha P^32^-dCTP (3,000 Ci/mmole; PerkinElmer, Wellesley, MA), 0.2 mM of each dNTP, and 1 U of *Taq* DNA polymerase (Bangalore Genei^TM^, Bangalore, India) in a standard buffer supplied by the vendor. PCR was carried out in a Thermal Cycler PTC150 (MJ Research, Watertown, MA) under the following conditions: an initial denaturation at 95 °C for 2 min was followed by 35 cycles at 95 °C for 30 s, 55-72 °C for 30 s, 72 °C for 1 min with a final extension at 72 °C for 5 min. Following PCR-SSCP, the gels were dried and subjected to Phosphor Image analysis (Fuji, Kanagawa, Japan). For sequencing, PCR was carried out as aforedescribed but without including alpha P^32^-dCTP in the reaction mixture. PCR amplified products were purified using Auprep^TM^ PCR Purification Columns (Invitrogen, Delhi, India) and sequenced on an ABI PRISM A310 automated sequencer (PE Biosystems, Foster City, CA). Since we did not identify the *MYOC* Gln48His mutation reported from India earlier [19] in our samples by PCR-SSCP analysis, we used allele-specific PCR to screen the samples for the presence of this mutation. The sequence of the common forward primer for allele-specific PCR is as follows: 5'-TGC AAT GAG GTT CTT CTG TGC ACG-3'. The sequences of the reverse allele-specific primers are as follows: 5'(mutant allele)-GAC TGG CCA CAC TGA AGG TAT AA-3' and 5'(wild-type allele)-GAC TGG CCA CAC TGA AGG TAT AC-3'. A 190 bp amplicon was generated with common forward and wild-type/mutant allele primers. Following detection of this mutation in patient samples, PCR products were sequenced to confirm the presence of the mutation. The *MYOC*-83G>A promoter polymorphism was studied by PCR-SSCP analysis. Primer sequences for this polymorphism are as follows: forward 5'-CAG CCT CAC GTG GCC ACC TCT GTC-3' and reverse 5'-AGG CCC AAA GCT GCA GCA ACG TGC-3'. These primers amplify a 196 bp amplicon. In order to see whether there could be a common origin of a specific *CYP1B1* mutation, all patients with the mutation were genotyped with four microsatellite markers from the *CYP1B1* candidate region and three intragenic *CYP1B1* single nucleotide polymorphisms (SNPs). The order of markers with respect to *CYP1B1* is as follows: D2S177-D2S1346-*CYP1B1*-D2S2974-D2S2331 (taken from the UCSC Genome Bioinformatics site).

**Table 1:** Details of primer sets used in the PCR-SSCP analysis of the *CYP1B1*, *MYOC*, *OPTN*, and *OPTC* genes.

Gene	Exon	Primer sequence (5'-3')	Amplicon size (bp)	AT (°C)
*CYP1B1*				
	2	CY2AF:TGTCTCTGCACCCCTGAGTGTCA	264	67
		CY2AR:GAGGTGAGCCGCCTGGCCCAC		
	2	CY2BF:TGCTGAGGCAACGGAGGCGGCA	211	68
		CY2BR:TGCACCAGGGCCTGGTGGATGG		
	2	CY2CF:TCCAGATCCGCCTGGGCAGCTG	262	67
		CY2CR:CTCAGCACGTGGCCCTCGAGGA		
	2	CY2DF:CAGCATGATGCGCAACTTCTTCAC	254	64
		CY2DR:GCGCCCGAACTCTTCGTTGTGGC		
	2	CY2EF:TGCCGCTACAGCCACGACGACC	261	64
		CY2ER:GAGGATAAAGGCGTCCATCATGTCG		
	2	CY2FF:GACAAGTTCTTGAGGCACTGCGAA	267	66
		CY2FR:TCAGAGGAGAAAAGACCTGGCCCA		
	3	CY3AF:GCTCACTTGCTTTTCTCTCTCCAC	255	60
		CY3AR:CCACAGTGTCCTTGGGAATGTGGTA		
	3	CY3BF:TCACTATTCCTCATGCCACCACTG	277	60
		CY3BR:TGAGCCAGGATGGAGATGAAGAGA		
	3	CY3CF:AAAGGCGGTGCATTGGCGAAGAAC	291	60
		CY3CR:TTACTCCTCATCTCCGAAGATGTGA		
*MYOC*				
	1	TG1AF:AAACCTCTCTGGAGCTCGGGCA	248	60
		TG1AR:TATACTGGCATCGGCCACTCTGG		
	1	TG1BF:GCCTGCCTGGTGTGGGATGTGG	255	64
		TG1DBR:GGCAGCCTGGTCCAAGGTCAATTGG		
	1	TIG1CF:GGAGGCCACCAAAGCTCGACTCA	212	60
		TG1CR:TCTCTTCCTCCAGAACTGACTTGTC		
	1	TG1DF:CCAAACCAGAGAGTTGGAGACTGC	256	62
		TG1DR:AGCCATATCACCTGCTGAACTCAGA		
	2	TG2F:CTCAACATAGTCAATCCTTGGGCCA	244	60
		TG2R:GACATGAATAAAGACCACGTGGGCA		
	3	TIGR1DF:CAAGTATGGTGTGTGGATGCGAGA	205	64
		TIGR1R:GCTCCCCGAGTACACCACAGCA		
	3	TIGR1F:GCCAAGCTTCCGCATGATCATTG	231	60
		TIGR1DR:GTGCCAACTGTGTCGATTCTCCA		
	3	TIGR2DF:CCTTCTAAGGTTCACATACTGCCTAG	270	66
		TIGR2DR:AATGGCACCTTTGGCCTCATCGGTG		
	3	TIGR3F:ACATTGACTTGGCTGTGGATGAAG	267	64
		TIGR3R:ATGGGATGGTCAGGGTCTTGCTG		
	3	TIGR4F:ACCTCAGCAGATGCTACCGTCAA	216	64
		TIGR4R:CCATTGCCTGTACAGCTTGGAGG		
*OPTN*				
	4	OPTN4aF:AATCGCCAATGGGTTTGTGGGA	198	64
		OPTN4aR:ACGTGTCCAGGTTTGGGTGGGC		
	4	OPTN4bF:AGGAGGACAGCCCCAGTGAAAGCAC	193	72
		OPTN4bR:AGGGATGGCATTTCTTGCAGGCCCA		
	5	OPTN5F:CACTTTCCTGGTGTGTGACTCCATC	280	64
		OPTN5R:AAAAAACAACATCACAATGGATCGGTC		
	6	OPTN6F:CAGCCTTAGTTTGATCTGTTCATTCAC	284	60
		OPTN6R:CCAGGGGAGGCTTTATAGTTTGCTC		
	7	OPTN7F:TGGAAGCTTCCTTGGGTTGCATGTC	275	65
		OPTN7R:AACATTTGACCTCCGGTGACAAGCA		
	8	OPTN8F:GGTTACTCTCTTCTTAGTCTTTGGAA	286	60
		OPTN8R:GTATCTTAATTATATCTCAGGAAAGCTG		
	9	OPTN9F:TTCTCTTAAAGCCAAAGAGAAAGTAAC	232	55
		OPTN9R:CACAAG ATTTGAATTCAGTGGCTGGA		
	10	OPTN10F:GTTTAATGTCAGATGATAATTGTACAGA	223	58
		OPTN10R:CTTTGTAAAAATGTATATTTCAAAGGAGG		
	11	OPTN11F:CGTAAAGGAGCATTGTTTATCCTCA	264	60
		OPTN11R:CAATCTGTATAAAAAGGCGATTCTCC		
	12	OPTN12F:GAAGGTTGGGAGGCAAGACTATAAG	224	60
		OPTN12R:CAACAGTTTCTGTTCATTACTAGGCTA		
	13	OPTN13F:CAGGCAGAATTATTTCAAAACCATTTCTAG	252	60
		OPTN13R:CAGGGCTGGCCTCGCTCAGCTGG		
	14	OPTN14F:TGCATTCATCTAGGTACTAAGTTCTG	229	60
		OPTN14R:TCTACGGCCATGCTGATGTGAGCT		
	15	OPTN15F:GTCTGCTCAGTGTTGTCATGTTTCG	243	60
		OPTN15R:GAATCCATTGTAGAGAATGAAGTGGAA		
	16	OPTN16F:CAAGTGAAACAAACACAACTGCCTG	227	60
		OPTN16R:CTGACATTTACCAACAGTTTTGGGGA		
*OPTC*				
	2	OP2aF:CACTCTGGAGAGCCTGTCCCTCAGA	227	64
		OP2aR:GAACTTCAAAGGAATCGCCTTCCCTG		
	2	OP2bF:CAGGAGACAGGGACAGCTTCTCTC	236	64
		OP2bR:CTCCCAGTGTCATGCAGGGAATGTA		
	3	OP3F:TTTGTGCAAAAGCTGGGCTACTGTG	230	60
		OP3R:GCCTATGACCTAGGGATATTGCGA		
	4	OP4F:GCCCCAGAGGCTAAAGAGATCTCC	265	64
		OP4R:CAGGGTGGCTGCATATGCCTGC		
	5	OP5F:AAAGATAGTGTGTTCTGGTTTCTCTC	296	64
		OP5R:GTGGTGGAGGTGATAGATAGTGGA		
	6	OP6F:CCAACAGGACCCACCAGCCTCCTA	209	64
		OP6R:CTGCTCCTGGTATCTAACTTCCATCC		
	7	OP7F:GGCAGAGCCTCTTGGTGAGGCTCA	276	64
		OP7R:GGCCCATGCCTGCATGGTCCTTG		

Note that primer sets CY2AF/CY2AR, OPTN4aF/OPTN4aR, and OPTN7F/OPTN7R work with 5% DMSO and 1.5 mM magnesium chloride. Primer sets CY2BF/CY2BR, CY2CF/CY2CR, CY2DF/CY2DR, and CY2EF/CY2ER work with 5% DMSO and 1 mM magnesium chloride. Primer set TG1BF/TG1DBR works with 1 mM magnesium chloride. The rest of the primer sets work with 1.5 mM magnesium chloride. Exon 1 of *CYP1B1*, exons 1, 2, and 3 of *OPTN*, and exon 1 of *OPTC* were not screened for mutations as they are non-coding. AT, annealing temperature.

## Results & Discussion

**Figure 1:**
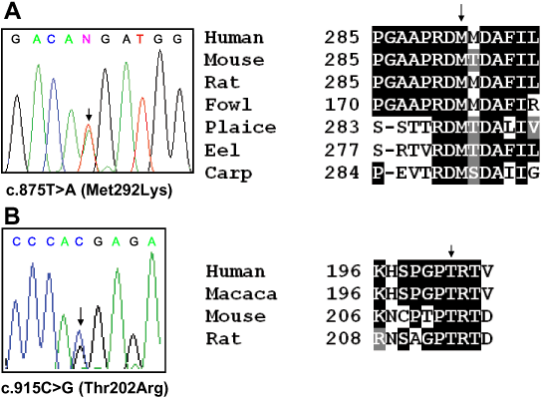
Mutation analysis of the *CYP1B1* and *OPTN* genes. **A**: The left panel shows a sequencing chromatogram from patient 119 who had a novel mutation c.875T>A (Met292Lys) in exon 2 of *CYP1B1*, and the right panel shows conservation of the methionine residue across species. **B**: The left panel presents a sequencing chromatogram from patient 30 who had a novel mutation c.915C>G (Thr202Arg) in exon 7 of *OPTN*, and the right panel shows conservation of the threonine residue across species. Arrows mark positions of nucleotide changes and conserved amino acid residues.

Analysis of the entire coding region of the *CYP1B1* gene in POAG patients revealed four different mutations (c.578C>T/Pro193Leu, c.685G>A/Glu229Lys, c.875T>A/Met292Lys, and c.1103G>A/Arg368His) in 10.76% (27/251) of patients (Table 2). Three mutations; Pro193Leu, Glu229Lys, and Arg368His, have been reported earlier in PCG patients from different populations [25-27]. The fourth mutation, Met292Lys, is a novel one in exon 2 and fulfills the criteria of a mutation since the methionine residue is conserved across species from human to carp (Figure 1A) and was not found in 97 controls (data not shown). Only patients 119 and 179 were compound heterozygous for Met292Lys/Arg368His and Pro193Leu/Glu229Lys, respectively (Table 2). The remaining patients were heterozygous for the Pro193Leu, Glu229Lys, Met292Lys, and Arg368His mutations (Table 2). Melki et al. [2] found that 4.6% (11/236) of French patients with early-onset POAG have mutations in *CYP1B1*. They have reported the Glu229Lys mutation in a heterozygous state. Colomb et al. [27] found that 6.45% (2/31) of French PCG patients have the Glu229Lys mutation in a heterozygous state. Acharya et al. [20] observed that 4.5% (9/200) of patients with JOAG from the eastern Indian state of West Bengal have mutations in *CYP1B1*. The Glu229Lys and Arg368His mutations were found in a heterozygous state, and these mutations were not present in 100 ethnically matched controls [20].

**Table 2:** Previously known and novel mutations detected in the *CYP1B1*, *MYOC*, and *OPTN* genes in primary open-angle glaucoma patients.

Patient (Age of onset/ diagnosis)	Location of mutation			IOP (RE:LE)	CD (RE/LE) ratio and other clinical details
	*MYOC*	*CYPIBI*	*OPTN*		
4 (53/55)	-	Arg368His/+	-	13/12	0.9/0.95 BE-not done because of poor vision
7 (49/50)	-	Arg368His/+	-	22/23	0,6/0.7; RE-upper arcuate defect LE-loss of sensitivity in the lower arcuate region
27 (56/57)	-	Glu229Lys/+	-	14/38	0.5/0.5; BE- reduced sensitivity upper arcuate defect
30 (52/53)	-	-	Thr202Arg*/+	28/22	0.8/0.4; RE-lower arcuate defect with nasal step, LE-loss of sensitivity in the lower arcuate region
37 (47/49)	-	Arg368His/+	-	38/26	0.5/0.3; RE-upper arcuate defect, LE-dense upper and lower arcuate detect going in for tubular vision
33 (46/46)	-	Arg368His/+	-	22/22	0.5/0.6; LE-loss of sensitivity in the lower arcuate region
44 (63/70)	Gln48His/+	-	-	43/48	0.9/0.9S BE-not done because of poor vision
54 (55/56)	-	Glu229Lys/+	-	19/20	0.8/0.7; NTG* BE-upper arcuate defect
61 (43/45)	-	Glu229Lys/+	-	17/18	0.5/0.6; NTG, BE- developing upper arcuate defect
66 (48/53)	-	Glu229Lys/+	-	22/28	0.8/0.9; RE-dense upper and lower arcuate scotoma going in for tubular vision, LE-PL-ve
68# (64/65)	Gln48His/+	-	-	28/32	0.6/0.5; RE-developing lower arcuate scotoma, LE -reduced sensitivity in lower arcuate region
69 (48/55)	-	Arg368His/+	-	21/32	0.9/0.9; BE-not done because of poor vision
71 (47/52)	-	Glu229Lys/+	-	25/23	0.8/0.9; BE-not done because of poor vision
79 (45/46)	-	Glu229Lys/+	-	28/32	0.5/0.6; BE-developing scotoma in the superior arcuate region
111 (50/53)	-	Arg368His/+	-	28/27	0.8/0.7; RE-upper arcuate scotoma, LE -reduced sensitivity in upper arcuate region
112 (62/64)	-	Glu229Lys/+	-	42/39	0.9/0.9; BE-dense upper and lower arcuate scotoma going in for tubular vision
119 (55/57)	-	Arg268His/	-	48/32	0.7/0.7; RE-not done because of poor vision, LE-dense tubular scotoma sparing the central vision
			Met292Lys*	-	
120 (55/63)	-	Met292Lys*/+	-	43/48	0.8/0.9; BE-not done because of poor vision
121 (52/60)	-	Met292Lys*/+	-	48/52	0.9/0.9S BE-not done because of poor vision
122 (55/57)	-	Met292Lys*/+	-	42/33	0.8/0.9; BE-not done because of poor vision
131 (57/58)	-	Glu229Lys/+	-	48/323	0.8/0.8; RE-not done due to poor vision, LE-isolated dense scotoma in (he inferior arena It region with nasal stepping
168 (45/47)	-	Glu229Lys/+	-	28/32	0.9/0.9; RE-dense upper and lower arcuate scotoma going in for tubular vision, LE-PL-ve
170 (48/49)	-	Arg368His/+	-	38/32	0.8/0.9; RE-dense upper and lower arcuate scotoma going in for tubular vision LE-isolated dense scotoma in lower arcuate region with nasal stepping
179 (50/52)	-	Glu229Lys/	-	28/32	0.7/0.7; RE-dense upper arcuate scotoma., LE-PL-ve
			Pro193Leu	-	
182 (59/79)	-	Prol93Leu/+	-	26/24	0.6/0.7; BE- developing upper arcuate defect
199 (55/57)	-	Glu229Lys/+	-	38/22	0.8/0.9; BE-dense upper and lower arcuate scotoma going Lit for tubular vision with macular sptepping
202 (50/53)	-	Arg368His/+	-	16/14	0.6/0.7; NTG, BE- developing lower arcuate defect
226 (69/72)	-	Arg368His/+	-	10/14	0.7/0.6; NTG, BE- developing upper and lower arcuate defect LE-isolated scotoma in die upper arcuate region
235 (62/63)	-	Glu229Lys/+	-	18/12	0.7/0.4; NTG* RE-doublc arcuate scotoma with nasal stepping
237 (69/70)	-	Glu229Lys	-	28/26	0.9/0.9; BE-dense double arcuate scotoma going in for tubular vision

Shown are details of 30 primary open-angle glaucoma (POAG) patients with previously known and novel mutations in the *CYP1B1*, *MYOC*, and *OPTN* genes. Since the Glu229Lys and Arg368His mutations were found in normal controls with frequencies of 5% and 2%, respectively, these mutations might be polymorphic variants in our population. By excluding these mutations, the frequency of patients with mutations in three glaucoma-causing genes is 3.59% (9/251). In the table, * indicates a novel mutation; + identifies wild-type alleles; # indicates a family history of glaucoma; other cases are sporadic. The following abbreviations were used: normal tension glaucoma (NTG), right eye (RE), left eye (LE), both eyes (BE), perception of light negative (PL-ve), and cup to disc (CD). The age of onset and diagnosis are in years.

Vincent et al. [15] reported the Arg368His mutation in a heterozygous state along with the *MYOC* Gly399Val mutation in an east Indian/Guyanese family with early-onset glaucoma. Of 30/251 patients with mutations in our samples, 13 patients (5.2%) had the Glu229Lys mutation and 10 patients (3.98%) had the Arg308His mutation (Table 2). In order to see whether these mutations were present in normal controls, we screened 100 normal controls. The Glu229Lys and Arg368 mutations were found with frequencies of 5% and 2%, respectively, in normal controls (data not shown), suggesting that these mutations might be polymorphic variants in our population. The presence of the Arg368His mutation in controls is not surprising as Melki et al. [2] discovered 2.13% (1/47) of French controls with this mutation. The Glu229Lys and Arg368His mutations were the most common mutations in Indian PCG patients with frequencies of 16.22% (6/37) and 59.46% (22/37), respectively [28]. However, ethnically matched population screening of 140 chromosomes for the *CYP1B1* mutations showed 6.4% and 0.7% carriers for the Glu229Lys and Arg368His mutations, respectively [28]. The other known mutation, Pro193Leu, was not present in 100 normal controls (data not shown). In order to determine whether the Glu229Lys variant in 13 patients could have a common origin, all patients were genotyped with four microsatellite markers (D2S177-D2S1346-*CYP1B1*-D2S2974-D2S2331) and three intragenic *CYP1B1* SNPs. An absence of allele sharing for the markers in patients suggested that Glu229Lys has multiple origins (Table 3). A similar result was obtained for the Arg368His mutation (Table 3).

**Table 3:** Genotypes at four microsatellite markers flanking the *CYP1B1* candidate region and three intragenic *CYP1B1* single nucleotide polymorphisms in patients with Glu229Lys and Arg368His variants

Patient	Variant	D2S177	D2S1346	C.142C>G	c.355G>T	c.1294G>C	D2S2974	D2S2331
27	Glu229Lys	1 5	2 3	C G	G T	CC	1 2	2 4
54	Glu229Lys	4 4	1 2	GG	TT	CC	1 2	4 5
61	Glu229Lys	4 6	4 4	C G	GT	CC	2 2	3 6
66	Glu229Lys	2 5	2 4	CG	GT	CG	2 2	3 4
73	Glu229Lys	4 9	2 2	C C	GT	C G	2 2	4 7
79	Glu229Lys	4 5	3 4	GG	TT	CC	2 2	4 7
112	Glu229Lys	4 5	2 4	CG	GT	CC	1 2	7 8
131	Glu229Lys	4 5	2 5	C G	GT	CC	2 2	7 10
I6S	Glu229Lys	5 7	2 2	CG	GT	CC	2 2	4 7
179	Glu229Lys	3 3	3 4	CG	GT	CG	2 2	3 8
199	Glu229Lys	6 7	4 4	CG	GT	CC	2 2	3 4
235	Glu229Lys	7 9	2 3	CG	GT	CC	2 2	3 7
237	Glu229Lys	3 3	4 5	CG	GT	CG	2 3	3 7
4	Arg368His	6 8	2 6	CG	GT	CC	1 2	4 5
7	Arg368His	2 3	2 2	CC	GG	CG	2 2	1 3
37	Arg368His	3 3	1 1	CG	GT	CG	2 3	8 9
38	Arg368His	4 4	1 6	CC	GG	CG	2 2	4 5
69	Arg368His	3 4	1 1	CG	GT	C G	3 3	4 5
111	Arg368His	5 6	1 1	CG	GT	C G	2 3	4 5
119	Arg368His	4 5	2 4	CG	GT	CG	2 3	3 5
170	Arg368His	3 8	2 2	CG	GT	CG	2 2	4 7
202	Arg368His	3 4	1 4	CC	GG	CG	1 2	5 6
226	Arg36SIIis	3 8	4 4	CG	GT	C G	2 2	5 6

Each number under a microsatellite marker column represents an allele. As can be seen from the genotypes at four microsatellite marker loci, some patients with either of the variants did not share alleles. For example, patients 168 and 179 with Glu229Lys did not share alleles at D2S177, D2S1346, and D2S2331. Although single nucleotide polymorphisms (SNPs) are less polymorphic than microsatellite markers, patients 54 and 73 did not share alleles at c.142C>G. Similarly, patients 7 and 38 with Arg368His did not share alleles at D2S177, D2S1346, and D2S2331. However, alleles at three SNP loci were not useful to determine nonsharing of alleles for Arg368His. Overall, the results suggest multiple origins for both variants.

In addition to mutations, nine *CYP1B1* population variants and polymorphisms were also identified in our samples, including two novel SNPs (Table 4). It is interesting to note that c.142C>G (Arg48Gly) and c.355G>T (Ala119Ser) occurred at a high frequency and were in complete linkage disequilibrium (Table 4). Similarly, c.1294G>C (Val432Leu) and c.1347T>C (Asp449Asp) also occurred at a high frequency and were in complete linkage disequilibrium (Table 4). Interestingly, c.142C>G (Arg48Gly) and c.355G>T (Ala119Ser) always occurred with c.1294G>C (Val432Leu) and c.1347T>C (Asp449Asp) in patients. Similar results were obtained in JOAG patients and normal controls from the east Indian state of West Bengal (Table 5) [20]. The significance of this phenomenon is not clear at present.

**Table 4:** Polymorphisms and population variants observed in the *CYP1B1*, *MYOC*, *OPTN*, and *OPTC* genes in primary open-angle glaucoma patients and controls

**Gene**	**Polymorphism/ population variant**	**Exon**	**Intron**	**Frequency in**	
				**POAG patients**	**Controls**
*CYPIBI*	C.IVS1-120T		1	2/251 (0.80%)	-
	c.IVS 1-14-15delTC**		1	2/251 (0.80%)	-
	c.I42C>G(Arg48Gly)	2		165/251 (65.74%)	-
	c.355G>T(Alall9Ser)	2		165/251 (65.74%)	-
	c.729G>C (Val243Val)	2		2/251 (0.80%)	-
	c.l294G>C(Val432Lcu)	3		228/251 (90.84%)	-
	c.l347T>C(Asp449Asp)	3		228/251 (90.84%)	-
	c.l358A>G(Asn453Ser)	3		116/251 (46.22%)	-
	c. 14460G (Leu482Leu)**	3		1/251 (0.40%)	0/93 (0.0%)
*MYOC*	-83G>A (Promoter region)#			89/116(76.72%)	75/98 (76.53%)
	c.227G>A (Arg76Lys)#	1		89/116(76.72%)	74/97 (76.29%)
	c.366C>T(Glyl22Gly)	1		1/251 (0.40%)	-
	c.l041T>C(Tyr347Tyr)	3		9/251 (3.59%)	-
	c.l303T(Gly434Gly)**	3		1/251 (0.40%)	-
*OPTN*	c.412G>A (Thr94Thr)**	4		29/251 (11.55%)	23/50 (46%)
	c.603T>A (Met98Lys)*	5		20/251 (7.97%)	7/96 (7.29%)
	C.7120T (Alal34Ala)	6		1/251 (0.40%)	-
	IVS7-5T>C**		7	83/251 (33.07%)	11/50 (22%)
	1VS7-10G>A88		7	1/251 (0.40%)	0/50 (0.0%)
	IVS7+24G>A		7	36/251 (14.34%)	-
	c.l866G>A(Ser519Ser)**	15		2/251 (0.80%)	11/50 (22%)
*OPTC*	c.486C>T (Phel62Phe)**	4		1/251 (0.40%)	0/50 (0.0%)
	c.803T>C (Leu268Pro)	6		31/251 (12.35%)	-
	c.810G>A(Leu270Leu)	6		5/251 (1.99%)	-
	c.859G>A (Val287Met)**	7		1/251 (0.40%)	0/50 (0.0%)
	IVS2-150A**		2	1/251 (0.40%)	-

Shown are the frequencies of polymorphisms and population variants in the *CYP1B1*, *MYOC*, *OPTN*, and *OPTC* genes seen in primary open-angle glaucoma (POAG) patients and controls. In the table, * indicates a risk factor, ** notes a novel single nucleotide polymorphism (SNP), and # highlights two polymorphisms, -83G>A and Arg76Lys, that were detected in high frequencies in 116 patients and thus were not screened in the remainder of the 251 patients.

**Table 5:** Reported frequencies of known polymorphisms and population variants in the *CYP1B1*, *MYOC*, *OPTN*, and *OPTC* genes in primary open-angle glaucoma patients and controls from different populations

Gene	Polymorphism/ population variant	Frequency in population			Reference
		POAG (%)	Controls (%)	Population	
*CYP1B1*	C.IVS1-120T	23	22	Eastern India	Acharya etal [20].
	c.I42C>G(Arg48Gly)	43.5	39	Eastern India	Acharya et al. [20]
	c.355G>T(Alall9Ser)	43.5	39	Eastern India	Acharya et al. [20]
	c.729G>C (Val243Val)	1.27	4.26	France	Mclki etal [2].
	c.l294G>C(Val432Leu)	51	59	Eastern India	Acharya et al. [20]
	c.l347T>C(Asp449Asp)	51	60	Eastern India	Acharya et al. [20]
	C.1358A>G (Asn453Ser)	16.5	14	Eastern India	Acharya et al. [20]
*MYOC*	-83G>A (Promoter region)*	18	23	U.S.A.	Alwardet al [29].
		30	39	Japan	Suzuki et al. [41]
		73.2	68.62	Eastern India	Mukhopadhyy et al. [16]
	c.227G>A (Arg76Lys)	19	18.7	U.S.A.	Alwardet al [29].
		73.2	68.62	Eastern India	Mukhopadhyy et al. [16]
	c.366C>T(Glyl22GIy)	0.53	0.0	U.S.A.	Alwardetal [29].
	c.l041T>C(Tyr347Tyr)	5.4	7.7	U.S.A	Alward et al. [29]
*OPTN*	c.603T>A (Mel98Lys)	28.6	24.6	China	Leung et al. [36]
		13.6	2.1	U.S.A.	Rezaieetal [13].
		11	5.5	Eastern India	Mukhopadhyay et al. [21]
		4.1	0.0	South Indian state of Tamil Nadu	Sripriya et al. [22]
		16.9	5	Japan	Fuseet al [33].
		6.25	7	Germany	Weisschuh et al. [35]
		4.64	4.54	France	Mclki et al. [39]
		10.7	8.33	Morocco	Melki et. al. [39]
		13.33	13.78	Japan	Toda ct al [37].
		20.7	9.0	Japan	Alwardet al [38].
	c.712C>A (Alal34Ala)	1.75	0.92	Afro-Caribbean Jewish/Scottish/African Somalian	Willoughby et al. [42]
	IVS7+24G>A	10.9	4	China	Leung et al. [36]
*OPTC*	c.803T>C (Leu268Pro)	6.9	14.55	French-Canada	Friedman ct al [23].
	c.810G>A (Leu270Leu)	0.0	3.66	French-Canada	Friedman ct al [23].

We did not look for another *MYOC* promoter polymorphism at nt -1000 (-1000C>G; *MYOC.mt1*; asterisk) in our primary open-angle glaucoma (POAG) data set because Alward et al. [43] and Özgül et al. [44] have observed that it is not a risk factor for the development of glaucoma in patients from the U.S.A. or Turkey, respectively.

Analysis of the *MYOC* gene revealed that 2/251 (0.8%) patients had the Gln48His mutation in a heterozygous state. This mutation was not present in 100 normal controls (data not shown). No other mutation was detected in our samples. The Gln48His mutation has been detected in 4/200 (2%) POAG patients in a heterozygous state from West Bengal [19]. This mutation has also been detected in 5/200 (2.5%) PCG patients [19]. Previously, we detected a novel Pro274Arg mutation in a four-generation family with members affected with JOAG and POAG, and with one severely affected patient being homozygous for the mutation [18]. Overall, the frequency of *MYOC* mutations has been found to be 2-4% in different populations [14]. Kanagavalli et al. [17] noted 1.87% (2/107) of patients with POAG from the south Indian state of Tamilnadu who had mutations in the *MYOC* gene. However, Mukhopadhyay et al. [16] discovered a higher frequency (7.14% or 4/56) of POAG patients from West Bengal with *MYOC* mutations. This could be a statistical anomaly due to a small sample size or a true pattern representative of different geographic origins.

The *MYOC* promoter polymorphism at -83G>A was initially reported from Western countries ([14,29] Table 5). This polymorphism has been also observed in Hong Kong and the Philippines [30,31]. Alward et al. [29] suggested that it is unlikely to be a disease-causing mutation. Since this polymorphism was detected in many countries, we wanted to determine its prevalence in our patient and control samples. We did not find any significant difference in the frequency of -83G>A between POAG samples and controls (76.72% in POAG versus 76.53% in controls; Table 4). This is similar to the earlier report from West Bengal by Mukhopadhyay et al. [16]. Interestingly, -83G>A is in linkage disequilibrium with Arg76Lys both in patients and controls (Table 4 and Table 5), suggesting that the -83G>A polymorphism is not a risk factor for developing glaucoma in Indians. Taken together, our observations suggest that mutations in *MYOC* gene may not be a major factor in the appearance of the POAG phenotype in India.

Mutation analysis of the entire *OPTN* gene revealed that one patient (0.40%) was heterozygous for a novel mutation, Thr202Arg (c.915C>G) in exon 7 (Figure 1B, Table 2). This mutation fulfills the criteria of a mutation because it is not present in 93 controls (data not shown) and the threonine residue is conserved in human, macaque, mouse, and rat (Figure 1B). Sripriya et al. [22] recently screened the *OPTN* gene in 100 high tension glaucoma patients from another south Indian state, Tamilnadu, and did not detect any mutation. Mukhopadhyay et al. [21] screened the entire coding region of this gene and detected 6/200 (3%) POAG patients from West Bengal, who were heterozygous for the Arg545Gln mutation in exon 16. As observed in other populations [32-36], the present observation also suggests that *OPTN* mutations are rare in POAG patients. A total of eight mutations (Glu50Lys, c.691-692insAG, Arg545Gln, Ala336Gly, Ala377Thr, His26Asp, His486Arg, and Glu104Asp) have been reported in the *OPTN* gene from different populations, including the His486Arg mutation in a JOAG patient [13,21,32-36]. Interestingly, one of the three mutations, Arg545Gln (c.1944G>A), reported by Rezaie et al. [13], has been detected in similar frequencies in Japanese glaucoma patients and control subjects [37]. Alward et al. [38] commented that the Arg545Gln variation is likely to be a nondisease-causing polymorphism. With the Thr202Arg mutation identified in this study, the total number of *OPTN* mutations, excluding Arg545Gln, reaches eight.

Rezaie et al. [13] initially reported that Met98Lys (c.603T>A) is a risk-associated alteration (risk factor) for developing glaucoma. They found this variant in both affected individuals and controls, but it was more common in affected individuals than in controls (13.6% versus 2.1%). Alward et al. [38] and Fuse et al. [33] showed a significant association between Met98Lys and glaucoma in Japanese patients. On the other hand, Toda et al. [37] found similar frequencies of Met98Lys in Japanese glaucoma patients and controls. Interestingly, Met98Lys has been shown to be a polymorphic variant in German, French, and Moroccan patients [35,39]. Sripriya et al. [22] did not detect Met98Lys in 100 controls, although it was present in 7/170 (4.1%) POAG and 3/50 (6%) NTG patients from Tamilnadu. However, a statistical analysis did not show any significant correlation with clinical parameters [22]. In another study by Mukhopadhyay et al. [21], the frequency of Met98Lys was found to be 11% and 5.5% in POAG and controls from West Bengal, respectively. Sripriya et al. [22] found Met98Lys in 6% of NTG patients, whereas Mukhopadhyay et al. [21] failed to find it in NTG patients. In our dataset, the frequency of this variant was found to be similar in our POAG samples and controls (7.97% in POAG versus 7.29% in controls; Table 4), suggesting that it may not be a risk factor for developing glaucoma in Indian populations.

Mutation analysis of the *OPTC* gene did not detect any mutation in our POAG samples. Screening of the *OPTC* gene was carried out in POAG cases by Friedman et al. [23]. The Leu268Pro polymorphism, identified by Friedman et al. [23] in 6/87 (6.9%) of POAG/NTG patients and in 8/55 (14.55%) of controls (Table 5), was found in 31/251 (12.35%) of our POAG samples (Table 4). Another synonymous codon change Leu270Leu, identified by Friedman et al. [23] in 2/55 (3.66%) of controls (Table 5), was also detected in 5/251 (1.99%) of our POAG samples (Table 4).

In summary, 3.59% (9/251) of our POAG patients had mutations in the *CYP1B1*, *MYOC*, and *OPTN* genes. Two previously known *CYP1B1* mutations, Glu229Lys and Arg368His, were found in similar frequencies in POAG patients and controls, suggesting that these mutations might be polymorphic variants in our population. A similar situation exists for the *CYP1B1* Ala443Gly mutation, first reported by Melki et al. [2] in French patients, and was found to be a polymorphic variant in an Ethiopian population with a frequency of 7% [40]. No association was found between the *OPTN* Met98Lys variant and glaucoma. Mutations in *MYOC* and *OPTN* are rare in Indian POAG patients. This is the first study to document the prevalence of mutations in three glaucoma-causing genes in the same set of Indian POAG patients. Our study suggests that mutations in these genes are rare in Indian POAG patients.
